# Quality of Life, Activity Impairment, and Healthcare Resource Utilization Associated with Atrial Fibrillation in the US National Health and Wellness Survey

**DOI:** 10.1371/journal.pone.0071264

**Published:** 2013-08-12

**Authors:** Amir Goren, Xianchen Liu, Shaloo Gupta, Teresa A. Simon, Hemant Phatak

**Affiliations:** 1 Health Outcomes Practice, Kantar Health, New York, New York, United States of America; 2 Indiana University Department of Psychiatry, Indianapolis, Indiana, United States of America; 3 Pfizer Primary Care BU, Pfizer, Inc., New York, New York, United States of America; 4 Health Outcomes Practice, Kantar Health, Princeton, New Jersey, United States of America; 5 Global Pharmacovigilence and Epidemiology, Bristol-Myers Squibb, Pennington, New Jersey, United States of America; 6 Global Health Economics & Outcomes Research, Bristol-Myers Squibb, Princeton, New Jersey, United States of America; Iran University of Medical Sciences, Islamic Republic of Iran

## Abstract

**Objectives:**

This study builds upon current studies of atrial fibrillation (AF) and health outcomes by examining more comprehensively the humanistic burden of illness (quality of life, activity impairment, and healthcare resource utilization) among adult patients with AF, using a large, nationally representative sample and matched controls.

**Methods:**

Data were analyzed from the Internet-based 2009 US National Health and Wellness Survey. Outcomes were Mental and Physical Component Summary (MCS and PCS) and health utility scores from the SF-12, activity impairment, hospitalizations, and healthcare provider and emergency room (ER) visits. Patients with self-reported diagnosis of AF were matched randomly on age and gender with an equal number of respondents without AF. Generalized linear models examined outcomes as a function of AF vs. non-AF status, controlling for CHADS_2_ score, comorbidity counts, demographics, and clinical variables. Exploratory structural equation modeling assessed the above in an integrated model of humanistic burden.

**Results:**

Mean age of AF patients (1,296 from a total sample of 75,000) was 64.9 years and 65.1% were male. Adjusting for covariates, compared with non-AF patients, AF patients had lower MCS, PCS, and utility scores, greater activity impairment (rate ratio = 1.26), more traditional provider visits (rate ratio = 1.43), and increased odds of ER visits (OR = 2.53) and hospitalizations (OR = 2.71). Exploratory structural equation modeling analyses revealed that persons with AF experienced a significantly higher overall humanistic burden.

**Conclusions:**

This study highlights and clarifies the substantial burden of AF and its implications for preparing efficacious AF management plans to address the imminent rise in prevalence.

## Introduction

Atrial fibrillation (AF) is the most common cardiac arrhythmia. Approximately 3.03 million US adults currently have AF, projected to increase to more than 4.78 million by 2025 and over 7.56 million by the year 2050, affecting over 50% of adults aged 80 or older [1]. Clinically, AF varies in its manifestation and can be paroxysmal, persistent, or permanent, reflecting the extent to which a patient’s symptoms are transient vs. more chronic in nature [2]. As reported by the American Heart Association (2010), AF causes approximately 15% to 20% percent of strokes and is linked to a 50%–90% increased risk of death [3].

Strokes resulting from AF tend to be associated with poorer health outcomes than strokes that are not AF-related [4]. In particular, AF-related stroke patients are more likely to experience pneumonia, pulmonary edema, intracerebral hemorrhage, in-hospital mortality, and less favorable neurological prognoses [4]. The economic burden of AF is also substantial. The total direct cost of AF is approximately $8,705 per patient, as compared with matched non-AF controls [5]. Moreover, AF has been associated with indirect costs, in the form of lost work productivity and benefits [6]. Severity and frequency of AF symptoms have been linked to greater impairment [7].

AF affects patients’ health-related quality of life (HRQoL). Approximately one-third of AF patients may experience increased feelings of depression and anxiety, which can persist after six months [8]. Female sex, older age (>65 years), and co-morbid conditions are associated with poorer HRQoL in AF patients [9]. Newly diagnosed AF patients reported poorer HRQoL, as measured by SF-36, than the general population [10] and healthy controls [11]. Evidence is mixed with respect to the differential effects of AF subtypes (i.e., paroxysmal or permanent) on HRQoL [12]–[14].

The burden of AF may extend beyond patients themselves. Spouses of AF patients have reported lower HRQoL comparable to that of AF patients, with similar perceived impact of the disease on both groups [15].

Literature reviews indicate that perceived HRQoL among AF patients tends to be lower than that of matched controls or healthy members of the general community. However, only a handful of studies have examined HRQoL in a broad, more representative population of AF patients, and these few studies yielded inconsistent results and often had methodological flaws, such as inadequate sample sizes and the use of HRQoL measures of questionable validity, among other concerns [16], [17]. Moreover, at present, there are no studies examining the overall perceived humanistic burden of AF, which encompasses several domains typically examined as distinct (e.g., health status and work productivity) even though they are interrelated. The current study is intended to begin addressing these gaps in the extant literature.

The US National Health and Wellness Survey (NHWS) provides a demographically representative sample of the US population, as has been shown in comparisons with National Health and Nutrition Examination Survey (NHANES) and National Health Interview Survey (NHIS) data [18], [19]. The current study examined data from the 2009 NHWS, allowing for a large sample of AF patients and an equal number of healthy controls matched by age and gender, for comparison with the focal group. The NHWS incorporates HRQoL measures (described below) that have been validated across many populations. The data analyzed in the present research were collected prior to the official Food and Drug Administration (FDA) approval of newer oral anticoagulants.

The first objective of this study was to examine the incremental burden of illness (HRQoL and activity impairment) among adult patients with AF, compared with matched controls without AF, using patient reported outcomes from a national sample. The second objective was to assess incremental healthcare resource utilization associated with AF. The third objective was to evaluate the overall humanistic burden associated with AF, combining elements of the first and second objectives into a single model. These objectives build upon current findings in the literature by examining multiple outcomes in a single study, utilizing a broadly representative sample of respondents (both with and without AF), and providing a unified, exploratory model of overall burden.

## Methods

### Ethics Statement

All respondents of the NHWS provided informed consent electronically (in lieu of written consent, given that the survey was administered online), prior to answering any survey questions. All electronic consent forms were stored and associated with each respondent’s unique identifier. Respondents were identifiable only according to this unique, assigned identification number. The survey and procedure were approved by an institutional review board (Essex Institutional Review Board, Lebanon, NJ).

### Sample and Procedure

Data were obtained from the 2009 US NHWS, a sample of 75,000 adults (18+) in the US. The NHWS is a self-administered, annual, Internet-based survey developed and managed by Kantar Health to assess, across a fairly comprehensive number of health conditions, the individual demographic characteristics, medical history, healthcare utilization, attitudes, behaviors, and outcomes of a representative sample of respondents. The sample is drawn from an Internet panel maintained by Lightspeed Research (LSR), using points that can be accumulated and exchanged for prizes as an incentive for participation. Panel members are recruited through opt-in emails, partner panels, e-newsletter campaigns, banner placements, and affiliate networks, and they register via unique email addresses and passwords, completing an in-depth demographic profile. NHWS is one among many types of surveys for which members of the LSR panel can volunteer, and they are only invited to participate once per year in the NHWS, with participation across all surveys limited to a set number per year in order to maintain the integrity of the data. NHWS respondents are recruited based on quotas to mimic the gender, age, and ethnicity/race distribution reported by the US Census Bureau.

The current sample, pulled from among all 2009 US NHWS respondents, consisted of patients with a self-reported physician diagnosis of AF (“Have you ever experienced Atrial fibrillation?”, and “Has your Atrial fibrillation been diagnosed by a physician?”), as well as an equal number of non-AF controls matched randomly (1∶1) on age and gender.

### Measures

#### Independent variables

Demographic variables included self-reported age (continuous variable), gender (male or female), race/ethnicity (White or non-White), marital status (married/living with partner, divorced/separated/widowed, or single), education (some college education or more, or not college educated), income (< $25K, $25K to<$50K, $50K to<$75K, $75K+, or declined to answer), current cigarette smoker (yes or no), exercise vigorously for at least 20 minutes at least once in the past month (yes or no), alcohol use (yes vs. no, do not drink alcohol), employment type (employed or not), health insurance (yes or no), and BMI (underweight, normal, overweight, or obese).

The CHADS_2_ score for risk of stroke was calculated by adding 1 point each for congestive heart failure (C), hypertension (H), age >75 years (A), and diabetes mellitus (D), and 2 points each for prior stroke or transient ischemic attack (S*_2_*) [20]. A score of 0 was classified as low risk, 1 as moderate risk, and 2 or more as high risk of having stroke.

Comorbidity counts (zero, one, two, or three or more) were based on comorbidities used in the Charlson Comorbidity Index (CCI) [21], a measure of patient mortality risk, but excluding those risk factors used in CHADS_2_ scoring, as well as diabetes with end organ damage and moderate/severe liver disease. The comorbidities included in the count (some of which can be confounded with the presence of AF) were: chronic pulmonary disease, peripheral vascular disease, myocardial infarction, HIV/AIDS, metastatic tumor, lymphoma, leukemia, any tumor, moderate/severe renal disease, hemiplegia, mild liver disease, ulcer disease, connective tissue disease, and dementia.

#### Dependent variables

Health-related quality of life (HRQoL) was assessed using the physical (PCS) and mental (MCS) component summary scores from the SF-12v2, and health utilities (calculated from seven SF-12v2 items) [22]. A health utility score is a preference-based single index measure for health using general population values. Health utility scores have interval scoring properties and yield summary scores on a 0 to 1 scale [23]. PCS and MCS scores are normed to the US population (Mean = 50, SD = 10) and vary from 0 to 100, with higher scores indicating greater HRQoL.

Depression, pain, and sleep symptoms were also assessed. Patient-reported experience of depression (“have you experienced depression in the past twelve months”), pain (“have you experienced pain in the past twelve months”), and insomnia (“did you experience insomnia or difficulty falling asleep, difficulty staying asleep, or waking up too early 2 times a week or more?”) in the past 12 months was assessed by presence or absence of the condition (0/1). Prevalence of pain, as assessed in the NHWS, has been shown to be consistent with other sources [24]. Insomnia, as assessed in the NHWS, has been validated against symptoms experienced by patients diagnosed with insomnia [18]. The Whooley depression screener was also used to assess the experience of depression (presence vs. absence) [25].

Patients reported on healthcare resource use, for their own medical condition: their number of traditional healthcare provider visits and whether or not they had visited the ER or were hospitalized for their own medical condition during the 6 months prior to the survey.

Activity impairment was produced by a single item on the Work Productivity and Activity Impairment: General Health questionnaire (a validated instrument that also examines productivity loss related to work) as the percentage of impairment patients experienced due to health problems during daily activities in the past 7 days [26]. The WPAI is a validated instrument that also provides measures of productivity loss related to work; however, for the present study, only activity impairment was used, because it was assessed for all respondents (not only employed respondents) and serves as a more general measure of productivity loss.

### Statistical Analyses

Patients reporting diagnosis with AF (n = 1,297) were matched randomly, without replacement, using SAS 9.1 PROC SURVEY SELECT (random seed = 499812) to an equal number of controls (i.e., respondents who were not diagnosed with and had never experienced AF), such that both groups had exactly matching distributions in terms of age and gender. These matched AF and non-AF groups were used in all analyses, including multivariable models that controlled for possible confounds beyond the two variables used in the matching process.

Bivariate comparisons between AF and non-AF patients were performed with Chi-square tests for categorical variables and independent samples t-tests for continuous variables.

Multivariable comparisons between AF and non-AF patients were undertaken using generalized linear models (GLMs) with maximum likelihood estimation. Covariates included age, gender, race/ethnicity, education, household income, smoking status, body mass index (BMI), exercise, alcohol use, employment, health insurance, CHADS_2_, and comorbidity count.

Given the normal distribution of the HRQoL measures (MCS, PCS, and health utilities), linear GLMs were used. Because of the pronounced skew of the count-like activity impairment and traditional healthcare provider variables, GLMs were specified to incorporate a negative binomial distribution (with log link function), providing the best fit to the data. A multiplicative dispersion parameter adjusted standard errors to compensate for slight model underdispersion. Logistic regression models were run for dichotomous outcome variables, including ER visits, hospitalizations, and pain, depression, and sleep symptoms variables.

Structural equation modeling (SEM) utilizes covariances among observed (measured) variables to model and test the statistical fit of latent factors (unobserved variables hypothesized to account for multiple observed variables) and causal/non-causal relationships among them. An SEM analysis can help identify a measurement model, using confirmatory factor analysis (CFA) to examine the pattern of relationships between observed variables and the latent constructs they are hypothesized to reflect, as well as providing an estimate of the reliability of the measured variables included in the model. SEM can also represent a structural (or path) model, depicting the relationships hypothesized among observed and latent variables. This is conceptually similar to running a series of regression equations, with the possibility of examining multiple outcomes and predictors simultaneously within the same model [27]–[29].

The purpose of the SEM analyses was to expand upon the initial GLMs and to test a comprehensive model of the psychosocial burden of AF and the extent to which AF status is associated with patient-reported outcomes. As multiple measures of health outcomes were expected to correlate with each other, a latent variable framework was created to test whether a single measure might account well for the overall patient-reported psychosocial burden. A structural equation model was developed in two stages, to address the measurement model of psychosocial burden and then to test that model of burden and its association with AF status and covariates.

**Figure 1:**
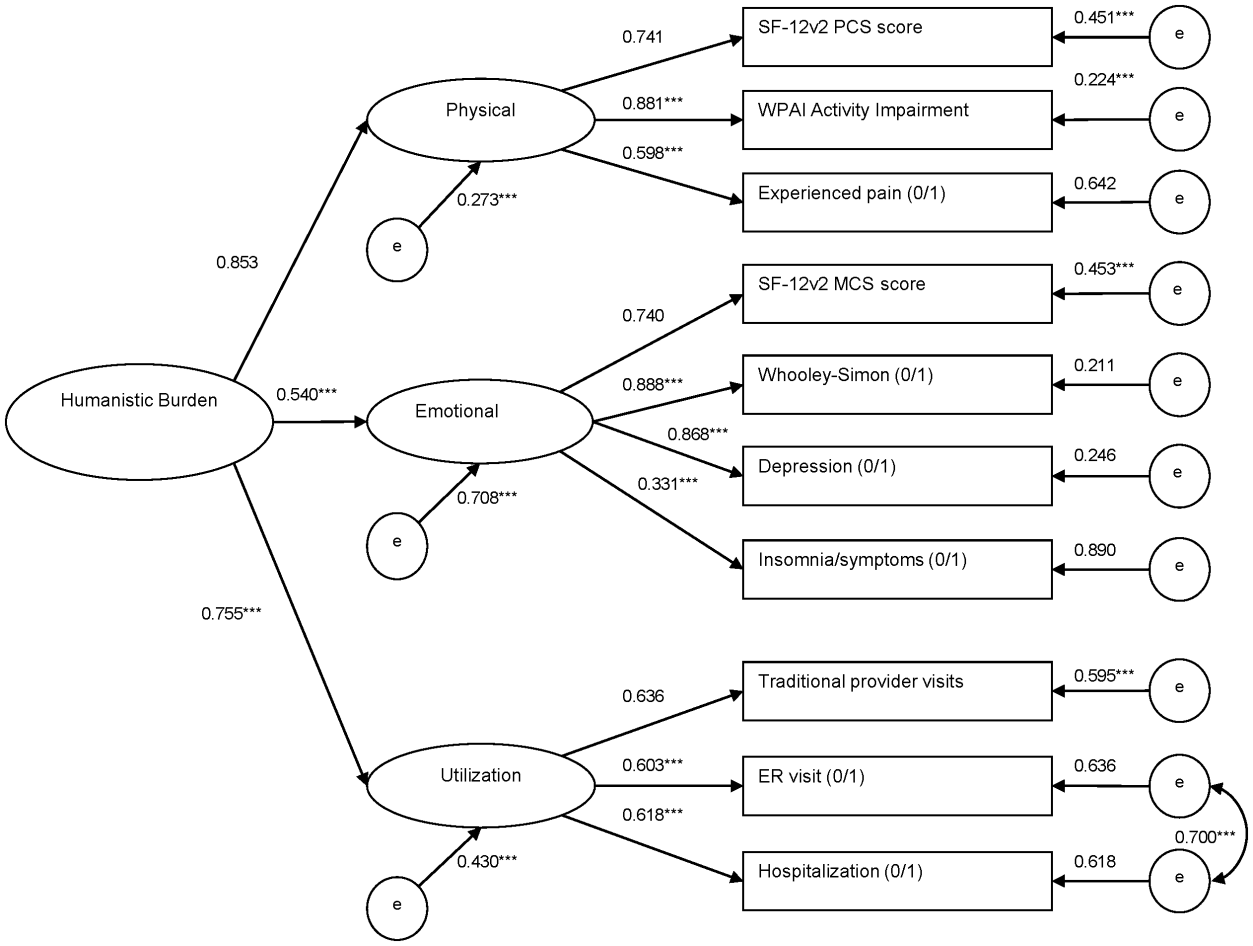
Overall Humanistic Burden: Confirmatory Factor Analysis. *Note.* Standardized estimates and residual variances are shown. Topmost indicators per factor set the scale. Dichotomous indicators include “(0/1)” in the name. *p<0.05. **p<0.01. ***p<0.001. No p-values are available for the scale setters and categorical residuals.

**Figure 2:**
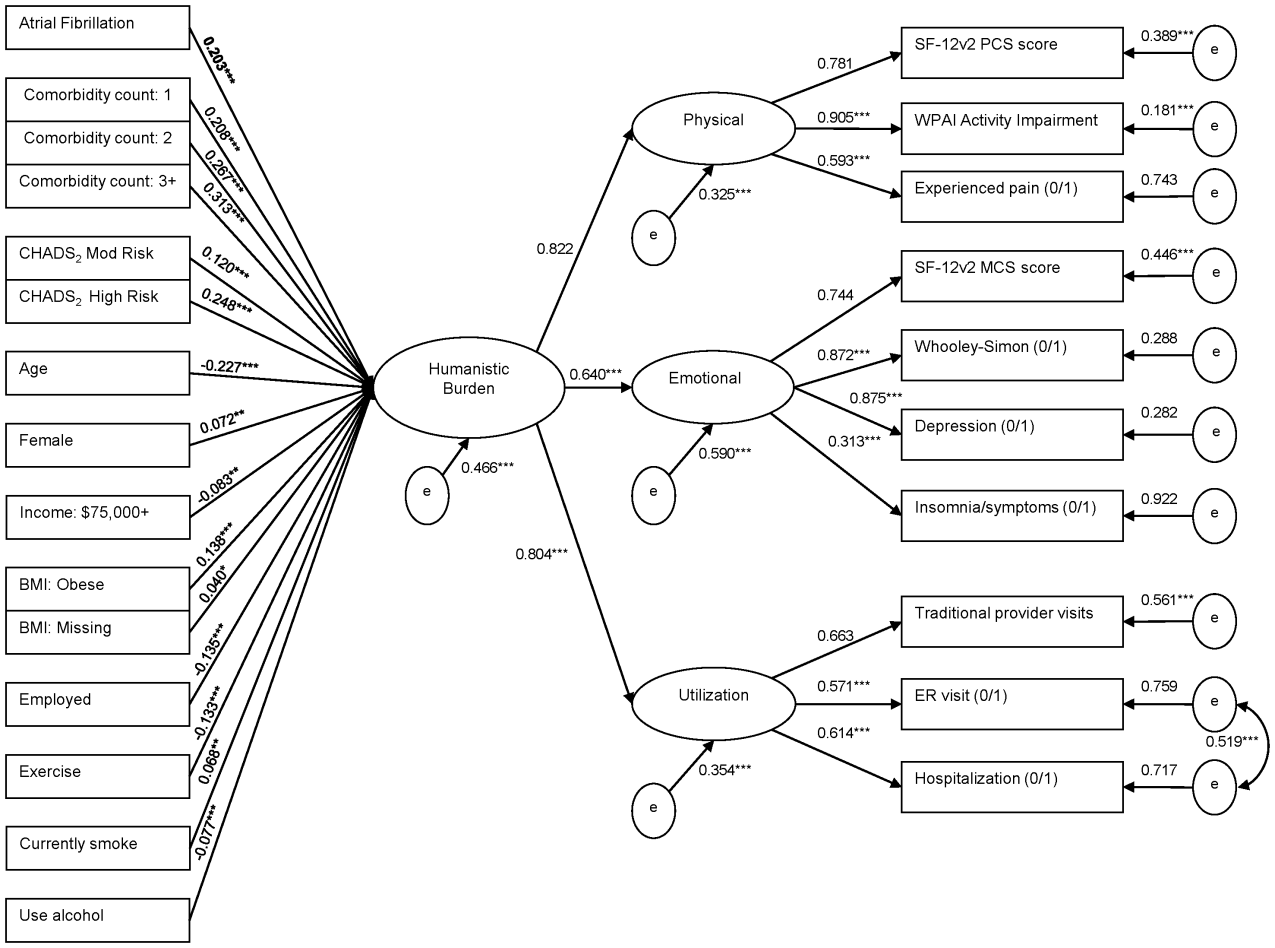
Overall Humanistic Burden as a function of AF, demographics, comorbidities, and behavioral factors. *Note.* Nonsignificant predictors of burden are not presented above: non-white; single; divorced/widowed/separated; college education or higher; household income: $25,000 to $49,999, $50,000 to $74,999, and decline to answer; uninsured; BMI: overweight. Reference groups include: non-AF matched controls, male, white, married/living with partner, less than college education, household income:<$25,000, insured, BMI: underweight/normal, unemployed, not exercising, not currently smoking, not using alcohol, comorbidity count of 0, and CHADS2 low risk. Standardized estimates and residual variances are shown. Topmost indicators per factor set the scale. Dichotomous indicators include “(0/1).” *p<0.05. **p<0.01. ***p<0.001. No p-values are available for the scale setters and categorical residuals.

An exploratory, comprehensive model of humanistic burden (measurement model) and the association between AF and covariates and humanistic burden (path model) was developed, as shown in Figures 1 and 2, on the basis of modifications to a model originally hypothesized to load on four components: functional, physical, and emotional impairment, and utilization of healthcare resources. These components were informed by the broad literature examining HRQoL, healthcare resource use, productivity impairments, and certain comorbidities as distinct health outcomes associated with the presence of various comorbid conditions (e.g., see studies by Lakkireddy et al. [30], Kirchhof et al. [31], and LaMori et al. [32]). The exploratory model, similar to the one depicted in Figure 1, was developed on the basis of conceptual face validity, using a priori categorization of individual outcomes within one of four relatively unique burden-related factors (noted above) that the items were thought to represent. The original categorization was modified slightly on the basis of model fit statistics. Modifications to both the measurement and subsequent path models were allowed only if considered conceptually sound and if indicated by modification indices forecasting a great improvement in model fit. Standardized estimates presented with the models indicate direction and strength of association between (a) indicators and their factors (factor loadings), and (b) factors and other variables in the model (path coefficients). Estimates are interpretable in a similar manner as correlation coefficients (range of −1 to +1, with 0 indicating no relationship).

Common fit indices include Chi-square test of model fit, Comparative Fit Index (CFI) and Tucker-Lewis Index (TLI), and Root Mean Square Error of Approximation (RMSEA) [33]–[35].

## Results

### Sample Characteristics

Among the 75,000 total US NHWS respondents, 1,297 (1.7%) reported having been diagnosed with AF. One AF patient could not be matched with a same-age corresponding non-AF control and was therefore dropped from analysis, resulting in two groups (AF and non-AF) consisting of 1,296 respondents each. Mean age was 64.9 years, and 65.1% were male.

Table 1 shows that AF patients vs. matched controls were more frequently white (92.8% vs. 88.0%, respectively), with a college education or higher (47.4% vs. 40.7%), obese (43.2% vs. 34.2%), and at high risk for stroke using CHADS_2_ scores (44.1% vs. 24.5%). AF patients vs. matched controls were also less frequently employed (25.9% vs. 33.6%).

**Table 1:** Characteristics of patients with AF and matched controls.

	Matched Control Group (N = 1,296)		AF Group (N = 1,296)		p-value
	Mean	SD	Mean	SD	
**Age**	64.86	12.17	64.86	12.17	NS
	**n**	**%**	**n**	**%**	
**Gender**					
Male	843	65.05	843	65.05	NS
Female	453	34.95	453	34.95	
**Race/Ethnicity**					
White	1140	87.96	1202	92.75	<0.0001
Black	70	5.40	27	2.08	<0.0001
Hispanic	32	2.47	31	2.39	0.8985
Other Ethnicity	54	4.17	36	2.78	0.0535
**Marital Status**					
Married	857	66.13	862	66.51	0.8354
Single	101	7.79	88	6.79	0.3261
Divorced/Separated/Widowed	338	26.08	346	26.70	0.7214
**Education**					
< College Education	768	59.26	682	52.62	0.0007
≥ College Education	528	40.74	614	47.38	
**Currently Employed**	435	33.56	335	25.85	<0.0001
**Having Health Insurance**	1185	91.44	1214	93.67	0.0300
**Daily exercise**	757	58.41	726	56.02	0.2184
**Currently smoke**	193	14.89	174	13.43	0.2844
**Use Alcohol**	747	57.64	775	59.80	0.2640
**BMI**					
Underweight	17	1.31	13	1.00	0.4626
Normal	321	24.77	224	17.28	<0.0001
Overweight	507	39.12	470	36.27	0.1337
Obese	443	34.18	560	43.21	<0.0001
Decline to answer	8	0.62	29	2.24	0.0005
**Income**					
Less than $25,000	220	16.98	221	17.05	0.9583
$25,000 to $49,999	424	32.72	418	32.25	0.8013
$50,000 to $74,999	268	20.68	270	20.83	0.9228
$75,000 and over	288	22.22	305	23.53	0.4267
Decline to answer	96	7.41	82	6.33	0.2769
**CHADS_2_**					
0 (Low Risk)	505	38.97	265	20.45	<0.0001
1 (Moderate Risk)	474	36.57	459	35.42	0.5393
≥2 (High Risk)	317	24.46	572	44.14	<.0001
**Comorbidities Count**					
0	938	72.38	698	53.86	<0.0001
1	239	18.44	362	27.93	<0.0001
2	73	5.63	132	10.19	<0.0001
≥3	46	3.55	104	8.02	<0.0001

### Health-Related Quality of Life

Unadjusted mean MCS, PCS, and utility scores were lower for the AF patients vs. non-AF controls (Table 2). Adjusting for covariates, AF patients vs. non-AF controls had significantly lower levels of MCS (least square [LS] means: 50.0 vs. 51.2, respectively; p<0.01), PCS (40.1 vs. 43.2; p<0.0001), and health utility scores (0.711 vs. 0.743; p<0.0001) (Table 3). Differences in both PCS (>3) and health utility (>0.03) scores exceed minimally important differences (MIDs) on those measures [36]–[38].

**Table 2:** Unadjusted health outcomes in patients with AF and matched controls.

	Matched Control Group (N = 1,296)		AF Group (N = 1,296)		p-value
	Mean	SD	Mean	SD	
**SF-12v2: Mental Component Summary**	51.56	9.69	49.66	10.92	<0.0001
**SF-12v2: Physical Component Summary**	44.76	11.24	38.62	12.01	<0.0001
**SF6D Utility Score**	0.757	0.140	0.697	0.143	<0.0001
**Overall Activity Impairment**	26.01	28.86	39.38	30.87	<0.0001
**Number of traditional provider visits**	4.82	5.39	8.19	7.83	<0.0001
	**n**	**%**	**n**	**%**	
**ER visits**	149	11.50	371	28.63	<0.0001
**Hospitalizations**	117	9.03	320	24.69	<0.0001
**Whooley Depression Screen**	330	25.46	422	32.56	<0.0001
**Experiencing Depression**	188	14.51	287	22.15	<0.0001
**Experiencing Pain**	456	35.19	612	47.22	<0.0001
**Experiencing Insomnia**	516	39.81	589	45.45	0.0037

**Table 3:** Adjusted health outcomes in patients with AF and matched controls.

	Matched Control Group (N = 1,296)		AF Group (N = 1,296)		p-value
	Least SquareMean	95% ConfidenceLimits	Least SquareMean	95% ConfidenceLimits	
**SF-12v2: Mental Component Summary**	51.23	(50.70,51.76)	49.99	(49.46,50.52)	0.0018
**SF-12v2: Physical Component Summary**	43.24	(42.70,43.78)	40.15	(39.60,40.69)	<0.0001
**SF6D Utility Score**	0.743	(0.736,0.750)	0.711	(0.704,0.718)	<0.0001
**Overall Activity Impairment**	25.44	(23.31,27.76)	32.49	(29.78,35.45)	0.0002
**Number of Traditional Provider Visits**	4.94	(4.71,5.18)	7.05	(6.74,7.37)	<0.0001

### Activity Impairment

Activity impairment was significantly higher for AF patients vs. non-AF controls (Table 2). Adjusting for covariates, AF patients vs. non-AF controls reported significantly higher activity impairment (LS means: 32.5 vs. 25.4, respectively; p<0.001) (Table 3).

### Resource Use

Resource use was considerably higher for AF patients than non-AF controls (Table 2). After adjustments, AF patients vs. matched controls reported more traditional healthcare provider visits in the past 6 months (LS means: 7.0 vs. 4.9, respectively; p<0.0001) (Table 3). The logistic regression models demonstrated significantly higher odds of an ER visit (Odds Ratio [OR]: 2.53; p<0.001) and hospitalization (OR: 2.71; p<0.0001) among AF patients vs. controls (Table 4).

**Table 4:** Adjusted healthcare resource use and depression, pain, and insomnia, in patients with AF and matched controls.

	Odds Ratio	Odds Ratio 95%Confidence Limits		p-value
**ER Visits**	2.532	2.020	3.173	<.0001
**Hospitalization**	2.710	2.122	3.460	<.0001
**Whooley Depression Screen**	1.204	0.992	1.461	0.0600
**Experiencing Depression**	1.338	1.061	1.687	0.0137
**Experiencing Pain**	1.330	1.121	1.580	0.0011
**Experiencing Insomnia**	1.183	0.998	1.401	0.0522

### Depression, Pain, and Sleep Symptoms

As shown in Table 2, depression, pain, and insomnia were more prevalent in AF patients than in their non-AF controls (all p-values <0.001). Adjusting for covariates, AF patients had significantly increased odds of experiencing depression and pain. They also had marginally significant (both p<0.07) increased odds of experiencing insomnia and Whooley-screened depression (Table 4).

### Modeling Overall Humanistic Burden

The full list of items considered for use as factor indicator variables consisted of HRQoL metrics (MCS, PCS, and health utilities), activity impairment, healthcare resource utilization (healthcare provider, ER, and hospital visits), and commonly experienced, burdensome comorbidities (diagnosed or screened depression, pain, and insomnia) with their intercorrelations examined to help inform the factor structure of the initial model. Due to problems with convergence and the high correlations between health utilities (an indicator for the functional factor) and MCS (for the emotional factor) and PCS (for the physical factor) scores, the functional factor and the health utilities item were dropped from subsequent models. Activity impairment was moved from the functional to the physical factor, due to conceptual similarities and a moderately high correlation with PCS. Some uncertainty about placement of the insomnia indicator was resolved following examination of the second model’s fit statistics, with a high modification index value suggesting a clear improvement in fit with insomnia as an indicator of the emotional factor. A particularly high modification index for the third model suggested that ER visits and hospitalizations be allowed to correlate; this was considered a reasonable modification on conceptual grounds, given that those variables loaded on the same factor and were likely to have correlated residual terms representing shared likelihood of visits due, for example, to different comorbidities.

Figure 1 shows the fourth, final model of overall humanistic burden, with a good model fit (χ^2^ test of model fit = 287.8, df = 31; CFI = 0.970; TLI = 0.956; RMSEA = 0.057, 90% CI = 0.051–0.063). The overall humanistic burden factor was represented in three first-order factors: physical, emotional, and healthcare utilization. Each of these factors in turn manifested as 3–4 observed measures specified previously. According to the standardized estimates, the physical factor was the most strongly representative of overall burden (0.853), followed by utilization (0.755), with the emotional factor being least representative (0.540).

The final measurement model (Figure 1) was incorporated into a path model (Figure 2), which shows the association between AF vs. non-AF matched controls and other variables on overall humanistic burden. This model had an adequate fit (χ^2^ test of model fit = 1056.5, df = 247; CFI = 0.897; TLI = 0.881; RMSEA = 0.036, 90% CI = 0.033–0.038). This was especially good for a newly specified multiple regression model, considering that the covariates explained 53.4% of the variance in overall humanistic burden. Supporting the findings of the separate multivariable models, AF status (AF patients vs. non-AF matched controls) was associated with a significant increase in overall humanistic burden. This effect was present even after controlling for other predictors of burden, many of which were significant independent contributors to greater burden: higher comorbidity counts, CHADS_2_ high and moderate risk scores, lower age, female gender, less than $75,000 income, obesity, unemployment, less exercise, smoking, and less alcohol use.

## Discussion

The current study found that in the US, AF is associated with reduced HRQoL, greater activity impairment, and increased healthcare resource utilization, adjusting for demographics, life styles, and comorbidities. AF patients were more likely than non-AF persons to report experiencing depression, pain, and insomnia. Unlike models examining individual outcome measures, the CFA model in the current study (Figure 1) assesses the extent to which humanistic burden is represented by its components, revealing that it is best manifested in physical burden, followed by healthcare utilization and emotional burden. Adjusting for covariates, AF vs. non-AF status was associated with a significantly increased overall humanistic burden of 0.2 points (on a possible scale of −1 to +1). The final path model (Figure 2) indicated that the burden of AF can be identified and measured concisely with a single latent factor, instead of having to fit multiple regression models for a number of individual outcomes. Moreover, the model allowed for a convenient, single-measure assessment of the total variance that could be explained in overall humanistic burden by AF status and the covariates (53.4%).

The present findings of lower HRQoL for AF vs non-AF patients are consistent with research showing that AF patients, compared with healthy controls, report reduced HRQoL across all domains of the SF-36 [11]. Moreover, the results for depression are in line with findings that a sizeable minority of AF patients experience elevated levels of depression and anxiety [8]. The higher healthcare resource utilization among AF patients is in accordance with research showing considerable direct [5] and indirect costs associated with AF [6].

The increased pain and insomnia found with AF are unique findings and fill a gap within the literature, highlighting the broad array of health outcomes that are associated with AF. Other studies have suggested a link between insomnia and certain cardiovascular conditions, but insomnia was viewed as a cause rather than consequence of comorbidity. Insomnia is a risk factor for acute myocardial infarction and coronary heart disease [39], [40], but its etiology remains a mystery [41]. Chronic pain and pain severity are also associated with insomnia [42], [43], so the current findings may reflect a pattern in which AF increases pain, which in turn increases likelihood of insomnia. Alternatively, AF may contribute to insomnia directly, or it may contribute to either insomnia or pain via adverse effects of treatment (e.g., bleeding risk associated with warfarin use, drug/food interactions, etc.). Future studies can investigate the likely causal sequences and interactions among AF, pain, and insomnia. Future research should not only attempt to replicate these findings but also assess whether HRQoL is improved by treatment regimens aimed at managing the incidence of pain and insomnia more effectively.

The current paper offers researchers a novel way to investigate the burden of AF and other diseases, by specifying a humanistic burden factor represented by an array of functional, physical, and emotional impairment-related measures. These different outcomes are often investigated individually, but the current study demonstrates that they are highly correlated within individuals and fit well within a single, higher-order model of burden. The current study also demonstrates that the presence of AF contributes significantly to the overall humanistic burden. This model is the first known attempt of its kind. Future research should extend this modeling to other disease areas and populations, both to help replicate and validate the model, and to establish simple, unified measures of disease burden that are comparable across conditions and populations.

### Strengths and Limitations

The present research has some notable strengths. A large, demographically diverse sample of AF patients was compared with an equal number of non-AF age and gender-matched participants. Additionally, a broad range of measures were employed to assess the key variables in this study, some of which have been well-validated for use within the population of interest. Finally, the current study provides a novel, unified examination of humanistic burden.

This study also has several limitations. Elderly persons may not be as comfortable using computers or have limited access to such technology, so these individuals may have been underrepresented, given the web-based survey used to collect study data. In contrast to the AF patient population often sampled for observational studies and randomized controlled trials, the current sample was representative of a younger AF patient cohort (<65 years). Moreover, although (consistent with prior research) AF was more prevalent among White vs. Black respondents, non-Hispanic Whites in the current study were, overall, overrepresented both among those with AF (92.8% vs. 84.7% according to the study by Go et al.) [44] and among the matched non-AF controls (88.0% vs. 76.2% according to 2010 US Census) [45]. This reflects underrepresentation of non-White respondents in particular age/gender subsets within the NHWS, suggesting potential issues with access among these respondents. Future research should look to employ alternative data collection strategies, such as telephone or face-to-face interviews, or paper-and-pencil surveys, to ensure solicitation of responses from an even more representative sample, which will likewise facilitate greater generalizability of findings to the broader population of AF patients.

Because self-report data were employed for all analyses, the findings are potentially limited by inaccuracies in participants’ recollections regarding medical diagnoses and other key study variables. In future studies, more objective information, including patients’ medical records, could corroborate self-report data.

The model of humanistic burden designed and tested in this study was derived from certain assumptions informed by the literature and driven by the data. However, alternative models, incorporating additional, fewer, or altogether different measures, within similar or different factor structures, may provide better fits to the data. For example, in the current study, we assumed that healthcare resource utilization, given that it is a health outcome commonly examined separately from HRQoL measures in the literature, ought to be considered as a distinct factor contributing to overall burden alongside physical and emotional components of HRQoL and comorbidities. However, it is possible that HRQoL is better modeled as mediating between conditions such as AF and resource use. Due to the lack of clear guidelines or justification in assuming that resource use is either a cause or consequence of HRQoL (or the consequence, along with HRQoL, of unmeasured third variables) on any consistent basis, it was decided for current purposes to assume the more neutral position that resource use occupies in the models tested.

Finally, a cross-sectional design precludes the ability to draw causal inferences from the data. Although multivariable analyses controlled for the influence of several potential confounding variables–including age and a number of comorbidities that were likely to co-occur with AF–it is still possible that some of the differences in outcomes between AF and non-AF controls were due to unmeasured variables. Since this study was based on a larger general health survey, the WPAI version that was used was not specific to AF, nor were the HRQoL or healthcare resource use measures. This, in addition to the cross-sectional design, limits inferences about the direction of causality between presence of AF and health outcomes. It would therefore be instructive to perform repeated measures or longitudinal analyses not only to replicate current findings but also to determine whether there are fluctuations in the perceived burden of AF patients over time.

### Conclusions

The current findings suggest that AF is associated with a substantial humanistic burden for patients. Appropriate treatment may help reduce this burden and improve HRQoL, especially if it effectively prevents symptom recurrence [46]. The present findings may also have important ramifications for the overall humanistic burden associated with this disease. The prevalence of AF is linked to age, with the condition largely afflicting elderly individuals. The number of individuals with AF is expected to increase markedly in the foreseeable future [1], [44]. In this context, the current findings are alarming and serve to underscore the urgent need to prepare efficacious AF management plans to reduce this imminent burden upon the healthcare system.
